# The Aqueous Extract of *Dacryodes edulis* (Burseraceae) Leaves Inhibits Cell Proliferation Induced by Estradiol on the Uterus and Vagina of Ovariectomized Female *Wistar* Rats

**DOI:** 10.1155/2020/8869281

**Published:** 2020-11-20

**Authors:** Marius Trésor Wego Kamgaing, Marie Alfrede Mvondo, Sylviane Laure Poualeu Kamani, Stéphane Minko Essono, Sylvie Lea Wansi Ngnokam

**Affiliations:** Department of Animal Biology, Faculty of Science, University of Dschang, P. O. Box 67 Dschang, Dschang, Cameroon

## Abstract

Proliferation is a cellular process strongly linked to the genesis of cancer. Natural substances with antiproliferative activities are currently potential alternatives in the treatment of cancers. *Dacryodes edulis*, for instance, is a medicinal plant traditionally used in the treatment of cancer. Scientific studies have reported the antioxidant activity of this plant. In addition, the presence of prostate cancer chemopreventive polyphenols was reported in *D. edulis* extracts. Therefore, this study was aimed to evaluate the effects of the aqueous extract of *D. edulis* leaves on cell proliferation induced by estradiol in ovariectomized female Wistar rats. In this regard, ovariectomized (OVX) rats were cotreated with estradiol valerate (E_2_V) (0.75 mg/kg) and the aqueous extract of *D. edulis* leaves. Control groups received either the vehicle (sham-operated animals and the OVX control), E_2_V (0.75 mg/kg) only, or E_2_V (0.75 mg/kg) and tamoxifen (10 mg/kg). Treatments were administered orally for 3 consecutive days, and animals were sacrificed thereafter. Epithelial heights of the uterus and vagina were assessed. Uterine levels of total cholesterol and estradiol were determined as well. Results showed that the aqueous extract of *D. edulis* leaves reversed the effects of estradiol as it reduced uterine weight (*p* < 0.05), uterine (*p* < 0.05), and vaginal (*p* < 0.001) epithelium heights. This antiproliferative effect of *D. edulis* was associated with reduced tissue (uterine) levels of estradiol (*p* < 0.001). These results suggest that the aqueous extract of *D. edulis* leaves could be a potential alternative treatment for proliferation-related diseases.

## 1. Introduction

Steroid hormones play an important role in regulating proliferation and protein synthesis of target cells. Estrogens particularly are known to stimulate a variety of biosynthetic processes, such as those of the breast and the uterus. In addition, nonsteroidal antiestrogens were found to antagonize many of the actions of estrogens through competitive estrogen receptor (ER) binding, inhibition of aromatase activity, and steroidogenesis [1, 2]. These antiestrogenic mechanisms could help reduce estrogenic response such as cell proliferation. Estrogen activities are mediated by two main isoforms of intracellular estrogen receptors: ER*α* and ER*β* [3, 4]. ER*α* is expressed in about 75% of diagnosed breast tumors [5], which can thus be treated with agents targeting estrogen-mediated signaling, such as antiestrogens [6, 7]. Estradiol has been recognized as the main hormone that stimulates the growth and development of cancer [8], especially estrogen-dependent cancers. They are the most prevalent hormone-related cancers in the world (50–80%) [9]. The introduction of adjuvant systemic therapy led to a significant improvement in postsurgical survival and a reduction in disease relapse, especially in women with early-diagnosed breast cancer and with positive ER tumors, who may receive endocrine therapy alone or in combination with cytotoxic therapy [10]. However, the use of these modern treatments was associated with substantial side effects [11, 12] and frequent recurrences [13]. Nowadays, the use of natural medicines as alternative treatments for cancer has greatly increased. The literature mentions the role of phytoestrogens in the treatment of breast cancer [14, 15]. Indeed, phytoestrogens were found capable of inducing biological responses, which may be able to modulate the actions of endogenous estrogens [16] through mechanisms including ER modulation, aromatase inhibition, antiangiogenesis, inhibition of hormone action, and modulation of hormone production [14, 17]. In order to elaborate effective strategies to control cell proliferation-related diseases such as estrogen-dependent cancers, research is increasingly focused on herbal medicines with traditionally reported anticancer properties and/or scientific evidence of antioxidant properties; as oxidative stress was found to promote cell proliferation [18–20] and to contribute to the initiation and progression of cancer [21].

*Dacryodes edulis* (Burseraceae) is a fruit tree widely spread in Cameroon. Its fruits are edible; meanwhile, the bark, leaves, stems, and roots are used as local medicine against some diseases such as anemia, dysentery, malaria, hypertension, cardiovascular diseases, and cancer [22–24]. The literature reports that extracts of fruits and leaves of *D. edulis* displayed antioxidant activities *in vitro* and *in vivo*, respectively [25, 26]. Phytochemical analysis performed on the aqueous extract of the stem bark and the fruitcake of this plant revealed the presence of secondary metabolites such as phenols, flavonoids, flavonols, and alkaloids [23, 24]. These compounds were reported to display antiproliferative activities *in vitro* [26, 27]. However, no scientific study reporting the *in vivo* antiproliferative effects of this plant was performed before. The present study therefore aimed at evaluating the ability of the aqueous extract of *D. edulis* leaves to inhibit cell proliferation induced by estradiol in ovariectomized rats. This effect was investigated on the uterus and vagina as both tissues are primary estrogen targets that rapidly respond to estradiol stimulation following a 3-day treatment in rats [28]. Qualitative and quantitative phytochemical analyses of this extract were performed to highlight bioactive compounds having antiestrogenic and antiproliferative activities. Uterine levels of total cholesterol and estradiol were assessed to illustrate the probable mechanism of action of the aqueous extract of *D. edulis* leaves.

## 2. Material and Methods

### 2.1. Chemicals

All the reagents were obtained commercially. Folin–Ciocalteu reagent, sodium carbonate, aluminium chloride, sodium nitrite, sodium hydroxide, sodium acetate, gallic acid, quercetin, chloroform, sodium hydroxide, chloric acid, iron chloride, acetic anhydride, sulfuric acid, and Mayer's reagent were purchased from Sigma-Aldrich (St. Louis, USA). Tamoxifen citrate was purchased from EuroGenerics (EG) Lab, Egis Pharmaceuticals Ltd, 1106 Budapest, Hungary. Estradiol valerate (Progynova 2 mg) was purchased from Bayer Pharma AG (Berlin, Germany). Diagnostic kits used for the estimation of total cholesterol were procured from Sigma-Aldrich (Stanford, Germany). All chemicals and reagents were of analytical grade.

### 2.2. Plant Collection and Preparation of the Aqueous Extract

#### 2.2.1. Plant Collection and Authentication

The leaves of *D. edulis* were collected in August 2018 at Dschang, the West Region of Cameroon, and authenticated in comparison with the botanical sample of Letouzez R. N. 4685 by Eric Ngansop Tchatchouang, a botanist at the Cameroon National Herbarium, where a voucher specimen has been deposited under the number 5552 SRF/CAM (YA). After collection, the leaves were shade-dried, then powdered with a mechanical grinder, passing through a sieve (11.2 mm with 7/16 standard mesh), and stored in an air-tight container at room temperature.

#### 2.2.2. Preparation of the Aqueous Extract and Determination of the Doses Tested

The extract was prepared following the recommendations of previous studies realized on the stem bark of this plant for phytochemical screening and medicinal potentials [23, 24]. Powdered leaves (300 g) of *D. edulis* were macerated in 3 L of distilled water for 48 h at room temperature. The supernatant was then filtered with Whatman paper no. 4 and dried in an oven at 40°C for two days. Twenty-nine grams (yield: 9.67%) of the aqueous extract was obtained and stored in an air-tight container at −20°C until use.

Four doses of this extract were administered to animals: 25, 50, 100, and 200 mg/kg BW. These doses have been extrapolated from previous studies realized on rats [25, 29]. Uhunmwangho and Omoregie [25] reported that the oral administration of 50 mg/kg of the methanol extract of *D. edulis* fruit induced antioxidant activity in rats. This dose was therefore divided by 2 to obtain the lowest dose of 25 mg/kg. The doses of 100 and 200 mg/kg were obtained by multiplying 50 mg/kg by 2 and 4, respectively.

### 2.3. Qualitative and Quantitative Analyses on Phytochemical Constituents

The qualitative analysis of phytochemical constituents was conducted following the methods described by Harbone [30] in a book entitled: “A guide to modern techniques of plant analysis”. The quantitative determination of total phenols, total flavonoids, and flavonol content was evaluated using the methods described by Ramde-Tiendrebeogo et al. [31], Chang et al. [32], and Almaraz-Abarca et al. [33], respectively. These phytochemical analyses were based on the appearance of different colours and the formation of precipitates or products in the final solution.

#### 2.3.1. Analysis of Alkaloids

A total of 0.1 gram of the aqueous extract was introduced in a test tube in the presence of 3 mL of hydrochloric acid (50% V/V). The solution was treated with 3 drops of Mayer's reagent, and the formation of a white precipitate indicated the presence of alkaloids [30].

#### 2.3.2. Analysis of Phenols

For phenol determination, 0.1 gram of the aqueous extract was solubilized in 3 mL of ethanol. The solution was treated with 3 drops of iron chloride III 10% (V/V), and the appearance of a blue-violet color indicated the presence of phenols [30].

#### 2.3.3. Analysis of Flavonoids

Few drops of 1% NH_3_ solution was added to 0.1 gram of the aqueous extract contained in a test tube. The appearance of a yellow color indicated the presence of flavonoid compounds [30].

#### 2.3.4. Analysis of Sterol and Triterpenoids

A total of 0.1 gram of the aqueous extract was solubilized in 3 mL of chloroform. A volume of 3 mL of acetic anhydride has been added, and the solution was frozen for 3 min. One drop of concentrated sulfuric acid was added. The presence of triterpenoid was confirmed by the appearance of a red-violated color, while that of sterols was indicated by a successive appearance of blue, green, red, and orange colors [30].

#### 2.3.5. Analysis of Tannins

For tannins identification, 0.1 gram of the aqueous extract was boiled in 20 mL of distilled water in a test tube and then filtered. The filtration method used here is the normal method, which includes a conical flask and filter paper. Three drops of 0.1% FeCl_2_ were added to the filtered samples and observed for a brownish green or a blue black color, which indicates the presence of tannins [30].

#### 2.3.6. Analysis of Saponins

Two grams of powdered samples of the plant was boiled together with 20 mL of distilled water in a water bath and filtered. A volume of 10 mL of the filtered sample was mixed with 5 mL of distilled water in a test tube and shaken vigorously to obtain a stable persistent froth. The frothing was then mixed with three drops of olive oil and observed for the formation of emulsion, which indicates the presence of saponins [30].

#### 2.3.7. Analysis of Anthocyanins

For the identification of anthocyanins, 0.1 gram of the aqueous extract was boiled in 5 mL of HCl (1% V/V). The presence of anthocyanins was traduced by the appearance of an orange color [30].

#### 2.3.8. Analysis of Anthraquinones

A volume of 4 mL of ether-chloroform (1 : 1 v/v) solution was added to 0.1 gram of the aqueous extract. The solution was treated with 4 mL of sodium hydroxide 10% (P/V). Anthraquinones were identified by the appearance of a red color [30].

#### 2.3.9. Quantitative Analysis of Total Phenols

Total phenolic content in the extract was determined by the modified Folin–Ciocalteu method [31]. The reaction mixture consisted of 200 *µ*L of extract, 200 *µ*L of 2N Folin–Ciocalteu reagent, and 400 *µ*L of 20% sodium carbonate solution. The mixture was stirred and incubated in a water bath at 40°C for 20 minutes. The experiment was carried out in triplicate. The absorbance was read at 760 nm. Total phenolic content was expressed as milligram gallic acid equivalent/gram extract (mgGAE/gE) using the equation obtained from a calibration curve of gallic acid (*y* = 1877.9*x* + 0.1142; *R*^2^ = 0.9989).

#### 2.3.10. Quantitative Analysis of Total Flavonoids

Total flavonoids were estimated using the aluminium colorimetric method described by Chang et al. [32]. In brief, 1500 *μ*L of distilled water and 30 *μ*L of sodium nitrite at 5% were added to 100 *μ*L of the extract. After 5 min of incubation at room temperature, 30 *μ*L of aluminium chloride (10%) and 200 *μ*L of sodium hydroxide (1 M) were added to the mixture. The experiment was carried out in triplicate. The absorbance was measured at 510 nm. Total flavonoid content was calculated as milligram quercetin equivalent/gram extract (mgQE/gE) using the equation obtained from the calibration curve (*y* = 300.75*x* + 0.0897; *R*^2^ = 0.9956).

#### 2.3.11. Quantitative Analysis of Flavonol Content

Flavonol content was determined according to the method of Almaraz-Abarca et al. [33]. In test tubes containing 1280 *μ*L of distilled water, 40 *μ*L of extract (2 mg/ml), 40 *μ*L of aluminium chloride (20%), and 40 *μ*L of sodium acetate (5%) were added. The experiment was carried out in triplicate. The absorbance was measured after 30 minutes at 415 nm. The flavonol content was calculated as milligram quercetin equivalent/gram extract (mgQE/gE) using the equation obtained from the calibration curve (*y* = 0.1872 x; *R*^2^ = 0.9734).

### 2.4. Experimental Animals

Juvenile female *Wistar* rats aged 2 months (100–120 g) were obtained from the breeding facility of the Research Unit of Animal Physiology and Phytopharmacology, University of Dschang (Cameroon). They were bred and kept under a standard soy-free rat diet in order to eliminate exposure to exogenous estrogenic compounds. All the animals were given free access to diet and water *ad libitum*. Animal handling and *in vivo* experiments were carried out after the approval of the research proposal by the scientific committee of the Department of Animal Biology, University of Dschang, in conformity with the European community guidelines EEC Council Direction 2010/63/EU of 22 September 2010 [34].

### 2.5. Study Design

Forty-two female *Wistar* rats were ovariectomized (OVX) as described by Mvondo et al. [35], and six other were subjected to a simple laparotomy (sham-operated). After two weeks of postsurgical treatments, the animals were randomly distributed into 8 groups of 6 animals each as follows:

1. SHAM: sham-operated animals, receiving distilled water (10 ml/kg)
2. [OVX]: Ovariectomized (ovx) rats receiving distilled water (10 ml/kg)
3. [OVX + E_2_V]: ovx rats receiving estradiol valerate (0.75 mg/kg)
4. [OVX + E_2_V + TAM]: ovx rats receiving estradiol valerate (0.75 mg/kg) and tamoxifen (10 mg/kg)
5. [OVX + E_2_V + AE25]: ovx rats receiving estradiol valerate (0.75 mg/kg) and the aqueous extract of *D. edulis* leaves at the dose of 25 mg/kg
6. [OVX + E_2_V + AE50]: ovx rats receiving estradiol valerate (0.75 mg/kg) and the aqueous extract of *D. edulis* leaves at the dose of 50 mg/kg
7. [OVX + E2V + AE100]: ovx rats receiving estradiol valerate (0.75 mg/kg) and the aqueous extract of *D. edulis* leaves at the dose of 100 mg/kg
8. [OVX + E_2_V + AE200]: ovx rats receiving estradiol valerate (0.75 mg/kg) and the aqueous extract of *D. edulis* leaves at the dose of 200 mg/kg

Animals were treated with the suboptimal dose of estradiol valerate (0.75 mg/kg) in reference to a previous work reporting the antiestrogenic effects of a medicinal plant [36]. The dose of tamoxifen (10 mg/kg) was chosen in reference to a previous study reporting the antiestrogenic activity of tamoxifen on breast cancer cells [37, 38]. Treatments were given orally for 3 consecutive days, and the animals were sacrificed thereafter under anesthesia (10 mg/kg diazepam and 50 mg/kg ketamine). The uterus and vagina were collected, free from fat tissues and weighed (only the uterus). The left horn of the uterus was homogenized and centrifuged at 3000 rpm for 15 minutes at 5°C. The resulting supernatant was collected and stored at −20°C for biochemical analysis. The right horn of the uterus and the entire vagina were fixed in 10% formalin for histological analysis.

### 2.6. Biochemical Analysis

Uterine total cholesterol levels were determined in each animal by an enzymatic method using commercial standard diagnostic kits purchased from SIGMA Diagnostics Ltd (1238 Budapest, Hungary).

Uterine estradiol levels were assessed by the ELISA test using a reagent kit (Mouse/Rat Estradiol ELISA) purchased from Calbiotech (El Cajon, California, USA). The absorbance of calibrators and specimen was determined using an ELISA microplate reader, the Multiskan ascent plate reader, purchased from MTX Lab Systems, Inc. (Bradenton, USA). The concentration (mIU/mL) was evaluated by means of a calibration curve established from the calibrators supplied with the kits.

### 2.7. Histological Analysis

Uterus and vagina epithelial heights were assessed from 5 *μ*m sections of paraffin-embedded uterine and vaginal tissues. Following the hematoxylin-eosin staining, epithelial heights were assessed on microphotographs using a computer connected to a light microscope provided by Olympus (Tokyo, Japan) where the image was transferred and analyzed with the Image J1.3 software.

### 2.8. Statistical Analysis

Data are expressed as mean ± standard error of the mean (SEM). Means were compared using one-way analysis of variance (ANOVA) followed by the Tukey post hoc test. GraphPad Prism, version 5.03, (for pharmacological tests) and Microsoft Excel 2007 (for phytochemical analysis) were used, and differences were considered significant at *p* < 0.05.

## 3. Results

### 3.1. Qualitative Phytochemical Analysis of the Aqueous Extract of *D. edulis* Leaves

Table 1 presents the phytochemical composition of the aqueous extract of *D. edulis* leaves. According to this table, the aqueous extract of *D. edulis* contains alkaloids, phenols, flavonoids, triterpenoids, tannins, saponins, anthocyanins, and anthraquinones. Sterols were not detectable in the extract.

### 3.2. Quantitative Phytochemical Analysis of the Aqueous Extract of *D. edulis* Leaves

**Figure 1:**
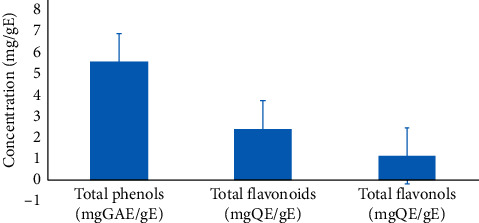
Quantitative analysis of phytochemical constituents of the leaves of *D. edulis*. GAE: gallic acid equivalent; QE: quercetin equivalent; E: extract.

The concentrations of total phenols, flavonoids, and flavonols in the aqueous extract of *D. edulis* leaves are presented in Figure 1. This figure shows that the aqueous extract of *D. edulis* contains a significant amount of total phenols (5.53 ± 0.07 mgGAE/gE), flavonoids (2.42 ± 0.28 mgQE/gE), and flavonols (1.17 ± 0.12 mgQE/gE).

### 3.3. Effects of Treatments on the Uterus

#### 3.3.1. Effects on Uterine Wet Weight and Histomorphology

**Figure 2:**
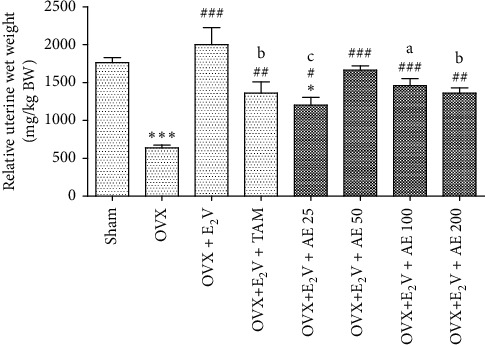
Effects of treatments on the relative uterine wet weight. SHAM: sham-operated animals; OVX: ovariectomized animals; E_2_V: estradiol valerate; TAM: tamoxifen; AE: aqueous extract of *D. edulis* leaves. Data are presented as mean ± S.E.M. (*n* = 6). ^*∗*^*p* < 0.05 and ^*∗∗∗*^*p* < 0.001 compared with SHAM; ^#^*p* < 0.05, ^##^*p* < 0.01, and ^###^*p* < 0.001 compared with OVX; and ^*a*^*p* < 0.05, ^*b*^*p* < 0.01, and ^*c*^*p* < 0.001 compared with OVX + E_2_V.

*(i) Effects on the uterine wet weight.* Compared with the SHAM control group, the relative uterine wet weight of ovariectomized animals decreased by 63.75% (*p* < 0.001) (Figure 2). Estradiol valerate (E_2_V) inversed this effect of ovariectomy as it increased the uterine wet weight by 212.88% (*p* < 0.001) compared with the OVX control. The coadministration of estradiol valerate with tamoxifen decreased the uterus relative weight by 32% (*p* < 0.01), compared with the OVX + E_2_V group. At tested doses, the aqueous extract of *D. edulis* leaves also inhibited the trophic effect of E_2_V by reducing the relative uterine wet weight compared with the OVX + E_2_V group.

**Figure 3:**
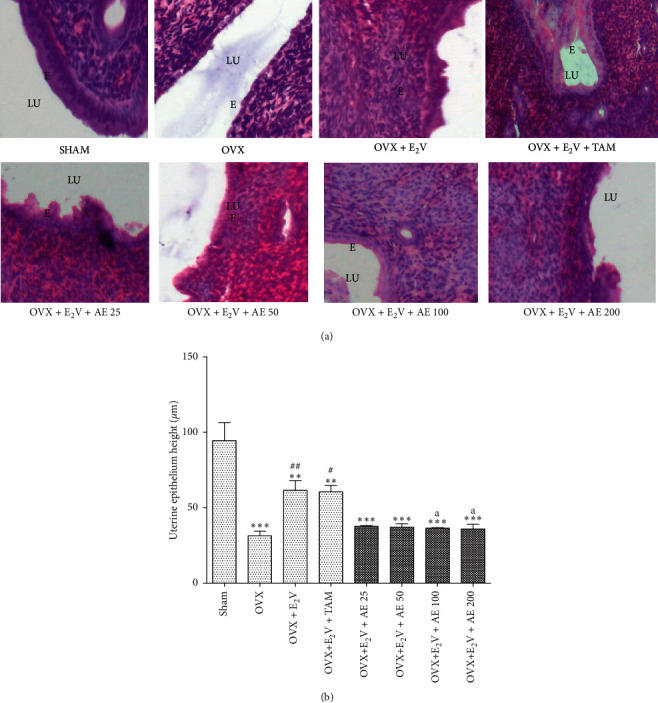
Microphotographs (H&E 400x) (a) and epithelial height (b) of the uterus after 3 days of treatment. SHAM: sham-operated animals; OVX: ovariectomized animals; E_2_V: estradiol valerate; TAM: tamoxifen; AE: aqueous extract of *D. edulis* leaves. Data are presented as mean ± S.E.M. (*n* = 6). ^*∗∗*^*p* < 0.01 and ^*∗∗∗*^*p* < 0.001 compared with SHAM; ^#^*p* < 0.05 and ^##^*p* < 0.01 compared with OVX; and ^*a*^*p* < 0.05 compared with OVX + E_2_V. LU: lumen; E epithelium.

*(ii) Effects on the uterine histomorphology*. The uterus of sham-operated animals was lined by a tall columnar epithelium (Figure 3(a)). The uterus of OVX animals presented a low cuboidal epithelium. Following treatment with estradiol valerate only, or in coadministration with tamoxifen, uterine epithelial cells were similar to those of sham-operated animals, but with a reduced thickness. The uterine epithelium of animals cotreated with estradiol valerate and the aqueous extract of *D. edulis* was similar to that of the OVX control group.

Figure 3(b) shows that ovariectomy decreased the uterine epithelial height by 57.14% (*p* < 0.001) compared with the SHAM control group. The daily administration of estradiol valerate increased this parameter by 51% (*p* < 0.05) compared with the OVX control. The coadministration of estradiol valerate with tamoxifen did not significantly affect the uterine epithelial height, which remained similar to that of the OVX + E_2_V group. At tested doses, the aqueous extract of *D. edulis* leaves inhibited the tropic effect of estradiol valerate by reducing (*p* < 0.05) the uterine epithelial height, with significant effects at doses of 100 (46.6%) and 200 (41.4%) mg/kg, compared with the OVX + E_2_V group.

#### 3.3.2. Effects on Uterine Levels of Cholesterol and Estradiol

**Figure 4:**
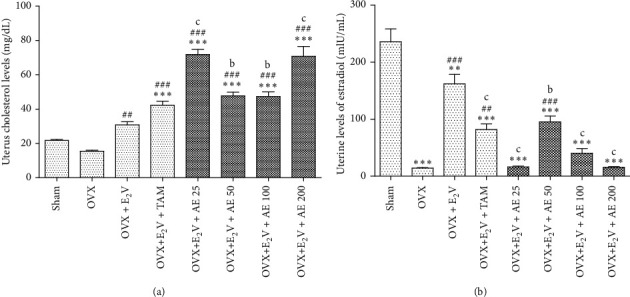
Effects of treatments on uterine levels of total cholesterol (a) and estradiol (b). SHAM: sham-operated animals; OVX: ovariectomized animals; E_2_V: estradiol valerate; TAM: tamoxifen; AE: aqueous extract of *D. edulis* leaves. Data are presented as mean ± S.E.M. (*n* = 6). ^*∗∗*^*p* < 0.01 and ^*∗∗∗*^*p* < 0.001 compared with SHAM; ^##^*p* < 0.01 and ^###^*p* < 0.001 compared with OVX; and ^*b*^*p* < 0.01 and ^*c*^*p* < 0.001 compared with OVX + E_2_V.

Compared with the SHAM control group, the uterine levels of cholesterol decreased by 29% in ovariectomized animals (Figure 4(a)). The oral administration of estradiol valerate inversed this effect of ovariectomy and increased this parameter by 99% (*p* < 0.05), compared with the OVX control. The coadministration of estradiol valerate with tamoxifen increased uterine levels of cholesterol by 37%, compared with the OVX + E_2_V group. The aqueous extract of *D. edulis* leaves further increased this parameter at tested doses, compared with the OVX + E_2_V group.

Uterine levels of estradiol reduced by 94% (*p* < 0.001) in ovariectomized animals, compared with the SHAM control group. The daily administration of estradiol valerate inversed this effect of ovariectomy as it increased this parameter by 1052% (*p* < 0.001), in comparison with the OVX control. The coadministration of estradiol valerate with tamoxifen reduced uterine levels of estradiol by 49.54% (*p* < 0.01), in comparison with the OVX + E_2_V group. A similar effect was observed in animals cotreated with E_2_V and the aqueous extract of *D. edulis* leaves at tested doses (25 mg/kg (90.1% induction, *p* < 0.001), 50 mg/kg (41.11% induction, *p* < 0.01), 100 mg/kg (75.13% induction, *p* < 0.001), and 200 mg/kg (90.5% induction, *p* < 0.001)), compared with the OVX + E_2_V group (Figure 4(b)).

### 3.4. Effects of Treatments on the Histomorphology of the Vagina

**Figure 5:**
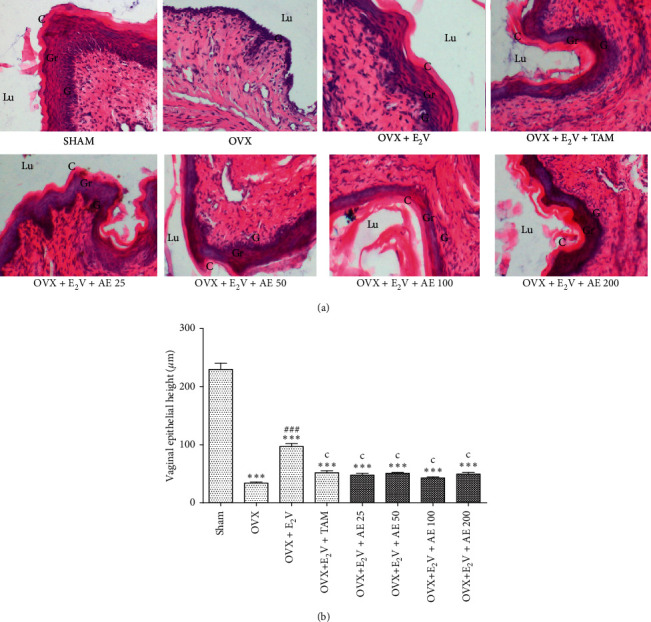
Microphotographs (H&E 400x) (a) and epithelial height (b) of the vagina after 3 days of treatment. SHAM: sham-operated animals; OVX: ovariectomized animals; E_2_V: estradiol valerate; TAM: tamoxifen; AE: aqueous extract of *D. edulis* leaves. Data from Figure 5(b) are presented as mean ± S.E.M. (*n* = 6). ^*∗∗∗*^*p* < 0.001 compared with SHAM; ^###^*p* < 0.001 compared with OVX; and ^*C*^*p* < 0.001 compared with OVX + E_2_V. Lu: lumen; G: stratum germinativum; Gr: stratum granulosum; C: stratum corneum.

The vaginal epithelium of the SHAM group exhibited three layers arranged, from the basal membrane to the lumen, as follows: stratum germinativum, stratum granulosum, and stratum corneum. Following ovariectomy, the vaginal epithelium was reduced to a single layer, the stratum germinativum. Following treatment with estradiol valerate, the vaginal epithelium was similar to that of the SHAM control, with the only difference that this epithelium was smaller than that of the aforementioned control. The coadministration of estradiol valerate with tamoxifen or with the aqueous extract of *D. edulis* leaves moderated the effect of estradiol valerate as it reduced the thickness of the vaginal epithelium (Figure 5(a)).

Figure 5(b) shows that ovariectomy decreased the vagina epithelial height by 85.21% (*p* < 0.001) compared with the SHAM control. Estradiol valerate inversed the effect of ovariectomy as it increased the vagina epithelial height by 185.29% (*p* < 0.001) compared with the OVX control. Tamoxifen inhibited the effect of estradiol valerate as it reduced the vagina epithelial height by 90.62% (*p* < 0.001), in comparison with the OVX + E_2_V group. The aqueous extract of *D. edulis* leaves induced the similar effect as it also decreased the vagina epithelial height at tested doses (25 mg/kg (92.45% induction, *p* < 0.001), 50 mg/kg (89% induction, *p* < 0.001), 100 mg/kg (95.32% induction, *p* < 0.001), and 200 mg/kg (91.34% induction, *p* < 0.001)), compared with the OVX + E_2_V group.

## 4. Discussion

Cell proliferation is the major precursor of cancer [18], such as estrogen-dependent cancers. These cancers are the most prevalent hormone-related cancers (50–80%) [9], whose genesis is stimulated by estrogens. These hormones are known to stimulate cell proliferation in estrogen-responsive targets such as the uterus and the vagina [28, 36, 39]. However, novel molecules are continuously being identified and developed from medicinal plants for better management of diseases in general and proliferative diseases in particular. These molecules in general are capable of inhibiting cell proliferation induced by estrogens [38]. To contribute to the quest for safer antiestrogenic and antiproliferative molecules, the *in vivo* antiestrogenic properties of the aqueous extract of *D. edulis* leaves were investigated.

Before evaluating the effects of the aqueous extract of *D. edulis* leaves on ovariectomized rats, a phytochemical analysis of that extract was performed. Results showed that the aqueous extract of *D. edulis* contains various secondary metabolites such as alkaloids, phenols, flavonoids, triterpenoids, tannins, saponins, anthocyanins, and anthraquinones. However, we observed the absence of sterols in this extract. These results are similar to those obtained by Ogboru et al. [23] in their research work in Nigeria in which they used the aqueous extract of the stem bark of *D. edulis*. In Ivory Coast, the fruitcake of *D. edulis* was also found to contain same groups of molecules [24]. However, contrary to these studies, sterols were not detectable in the aqueous extract of *D. edulis* leaves in the present research work. These results suggest that the harvest site and the part of the plant used have a direct impact on the presence of secondary metabolites in the plant. The quantitative analysis revealed that the aqueous extract of *D. edulis* leaves contains significant amounts of total phenols, flavonoids, and flavonols whose proportions were different (inferior) from those reported by Ogboru et al. [23] in Nigeria who worked with the stem bark and Ano-Aka et al. [24] in Ivory Coast who worked with the fruitcake of the same plant. These results suggest that the harvest site and the part of the plant used also have an impact on the amount of secondary metabolites in the plant. In addition, secondary metabolites such as anthraquinones, alkaloids, total phenols, flavonoids, and flavonols present in plants are reported to be antioxidant agents [23, 40, 41], which may act *in vivo* to prevent oxidative damage to DNA, proteins, and lipids, thus reducing cell proliferation and by extension the risk of cancer development [42]. To this regard, the literature reports that polyphenols such as tannins, saponins, and triterpenoids are bioactive compounds that possess antiproliferative and anticancer properties [43–45]. Maria Graça et al. [46] and Amoussa et al. [41] reported the antioxidant activities of phenols, flavonoids, and flavonols. Indeed, oxidative stress was found to promote cell proliferation [18–20], thus contributing to the initiation and the progression of cancer [21]. Therefore, plant extracts containing secondary metabolites with antioxidant activities could be efficient against proliferation-related diseases.

Results from the *in vivo* study showed that ovariectomy induced a significant reduction of the uterus relative weight. This result is in accordance with a previous report indicating that after 14 days of ovariectomy, there is an important reduction of endogenous estrogens inducing uterus atrophy [47]. In addition, the administration of estradiol valerate for 3 consecutive days was found to increase the uterus relative weight and the uterus and vagina epithelial heights. The augmentation of the uterus and vagina epithelial heights was in accordance with the histomorphology of these organs, which showed the hypertrophy of uterine epithelial cells and the stratification of the vaginal epithelium. These results are in accordance with previous reports realized with estradiol valerate on ovariectomized rats [28, 47]. The uterotrophic effect of estrogen has been attributed to at least two events: water imbibition and/or cell proliferation [48, 49]. Moreover, the literature reports that these trophic and proliferative effects on the uterus and the vagina are mediated by the estrogen receptor alpha (ER*α*) subtype [50, 51].

The daily coadministration of estradiol valerate with the aqueous extract of *D. edulis* leaves decreased the uterus relative weight and epithelial height of the uterus and the vagina. This effect could be due to the presence of flavonoids and flavonols in the aqueous extract of *D. edulis* leaves. Indeed, it has been shown that these compounds are capable of binding estrogen receptors [52, 53] and competing with estrogens for binding ERs [14, 54]. In addition, the literature reports that, in response to estrogen stimulation, uterine epithelial cells change from cubic to cylindrical [26, 55]. The bilateral ablation of the ovaries leads to atrophy of the uterus epithelium made up of cubic cells [28, 35]. Estradiol was also reported to be synthesized *in situ* in the uterus and to act in a paracrine manner to stimulate uterine growth [35, 56, 57]. In accordance with these literature data, our results showed that the uterine epithelium of ovariectomized animals (OVX) was made up of cubic cells, in comparison with SHAM control animals, in which this epithelium was rather cylindrical and might justify the reduction of the uterine epithelial height observed in the OVX group. The decrease of the uterine epithelium size in the OVX group was correlated with a decrease in uterine levels of total cholesterol. According to Zhao et al. [58], the *novo* synthesis of cholesterol in the uterus is stimulated by serum estradiol. Therefore, the bilateral ovariectomy, which was found to decrease serum levels of estradiol [35], would have decreased uterine cholesterol levels by lowering circulating estrogen levels. Although the aqueous extract of *D. edulis* leaves did not reverse the effect of estradiol valerate on uterine cholesterol levels, it reduced its effect on uterine steroidogenesis as it decreased uterine levels of estradiol. These results suggest that the aqueous extract of *D. edulis* leaves could have inhibited at least one stage of steroidogenesis, leading to reduced levels of estradiol. In addition, the aforementioned extract could be endowed with hypercholesterolemic properties as it reduced the use of uterine cholesterol for steroidogenesis. This effect could be due to the presence of flavonoids and anthocyanins in this extract as these phytochemicals were reported to increase HDL-cholesterol levels [59, 60]. Furthermore, the aqueous extract of *D. edulis* leaves induced hormesis-type responses on both cholesterol and estradiol levels in the uterus. Hormesis was defined as a dose-response relationship phenomenon characterized by low-dose inhibition and high-dose stimulation or inversely, leading to a U-shaped, an inverse U-shaped, or a J-shaped dose response [61, 62]. This dose-response revolution is not yet fully understood.

## 5. Conclusion

In summary, the present study showed that the aqueous extract of *D. edulis* leaves displayed antiproliferative activity as it inhibited the hypertrophy of uterine epithelial cells and the proliferation of vaginal epithelial cells, both events induced by estradiol valerate. This antiproliferative effect of the aforementioned extract may be mediated through the inhibition of tissue steroidogenesis as it decreased tissue levels of estradiol. These findings suggest that the aqueous extract of *D. edulis* leaves could be a promising alternative for the treatment of estrogen-dependent proliferative diseases.

## Figures and Tables

**Table 1:** Qualitative phytochemical analysis of the aqueous extract of *D. edulis* leaves.

Phytochemicals	Qualitative remarks
Alkaloids	+
Phenols	+
Flavonoids	+
Sterols	−
Triterpenoids	+
Tannins	+
Saponins	+
Anthocyanins	+
Anthraquinones	+

+ = detectable; − = not detectable.

## Data Availability

Data used to support the findings of this study are available from the corresponding author upon request.
